# Cardiotoxicity of plants in South Africa

**Published:** 2012-10

**Authors:** Pieter Van Der Bijl, Pieter Van Der Bijl

**Affiliations:** Division of Cardiology, Department of Medicine, Faculty of Health Sciences, Stellenbosch University and Tygerberg Academic Hospital; Emeritas professor, Department of Pharmacology, Faculty of Health Sciences, Stellenbosch University

The floral kingdom of southern Africa comprises well over 30 000 species of higher plants, many of which have the potential to be toxic to animals and humans.^1^,^2^ Livestock losses due to poisoning by plants have been significant over the years and have prompted extensive research efforts. Although there is a considerable body of information in the veterinary field, there is a paucity of published data on human poisoning in South Africa.^3^ While poisoning in livestock and humans is mainly accidental (e.g. confusing toxic with edible species, contamination of foodstuffs, for example by mycotoxins and other toxin-elaborating organisms, or perhaps overwhelmingly by medicinal use of plants in traditional medical practice), it may be deliberate.

The medicinal benefits of plants were recognised by the Egyptians and Romans who used an extract of *Urginea maritima* as diuretic, cardiotonic, expectorant and emetic.^4^ Furthermore, the medicinal value of *Digitalis purpurea* (foxglove, which contains cardiac glycosides) was reported by William Withering in 1785 after observing that patients with dropsy (cardiac failure) could be treated by using an extract from this plant.^5^

The use of plant-derived materials is widespread in the practice of traditional medicine in South Africa. It has been estimated that approximately 80% of the South African population consult traditional healers regularly.^6^ Traditional medicines (*muti*) are usually administered orally or as an enema by traditional healers. Laboratory analyses of *muti* have shown that these medicines often consist of aqueous plant materials, such as roots, bark stem or leaves, sometimes mixed with metallic salts, mushrooms and insects.^5^ Plant components are sometimes pulverised or sliced into small pieces, making botanical identification difficult or impossible.

Plants known to be toxic contain chemical constituents that can affect a wide range of organ systems; these have been documented in a number of publications. As far as the cardiovascular system is concerned, cardiac glycoside-containing plants are important in both lethal livestock and human poisonings.^3^,^7^ The cardiac glycosides, which are highly toxic and found in a number of plants, are usually phytochemicals consisting of an aglycone (structurally related to steroid hormones) linked to one or more sugar molecules. The aglycones of cardiac glycosides can be divided into two chemical groups, the cardenolides and bufadienolides.

The primary pharmacological effect of these cardiac glycosides is to inhibit the Na^+^/K^+^ ATPase exchanger, which increases intracellular Na^+^ concentration, thus reducing the Na^+^ gradient across the membrane and decreasing the amount of Ca^2+^ pumped out of the cell by the Na^+^/Ca^2+^ exchanger during diastole. Consequently, the intracellular Ca^2+^ concentration rises, thereby occasioning positive inotropy.

Cardiac glycosides have very narrow therapeutic indices and acute toxicity is most commonly associated with ingestion of plant material, although chronic toxicity may also sometimes be seen. In cases of acute intoxication, nausea, emesis and abdominal pain typically occur, as well as central nervous system effects including lethargy and weakness. Cardiac effects may manifest as nearly any type of dysrhythmia, and sudden death with few premonitory signs may occur.

Prominent among the garden-variety cardiac glycosidecontaining plants is *Nerium oleander* (oleander, *selonsroos*), which contains two cardenolides (oleandroside and neriin) characterised as potent cardiotoxins. *Thevetia peruviana* and *Thevetia thevetioides* (yellow oleander) contain thevetin, a potent toxic cardenolide that is widespread throughout the plant, but particularly concentrated in the fruits. Toxicity is retained when oleander is dried, and the plant material is very poisonous for both animals and humans. Ingestion of a single leaf by a child can be lethal. It is significant that the high toxicity of the plant is reflected in one of its colloquial names, the ‘Be-still tree’, and its fruit is referred to as the ‘Be-still nut’.

Other well-known cardiac glycoside-containing plants, such as *Digitalis purpurea* (foxglove), contain the cardenolide digoxin that is used therapeutically in the treatment of cardiac failure and supraventricular tachycardias. Its use in modern cardiology is currently limited, primarily because of its toxicity, narrow therapeutic index and the availability of superior treatment modalities.

*Acokanthera oppositifolia* (bushman poison bush, *boesmansgif*) sap contains cardenolides and has been used by the San people for applying to the tips of their hunting arrows. It is also used for treating headaches, snakebite, toothache, colds, anthrax and tapeworm infestation.^2^

The cardiotoxic bufadienolides present in *Drimia sanguinea* (sekanama, *slangkop*) and *Bowiea volubilis* (climbing potato, *knolklimop*) species have also been implicated in human poisoning.^2^ These plants are used by traditional healers as ‘blood purifiers’ and for treating a variety of conditions, such as headaches, oedema, infertility and bladder complaints.

It may be challenging to establish the diagnosis of plant poisoning in patients. It primarily relies on a history of ingestion of cardiotoxic plant material and/or suspicion generated by cardiac dysrhythmias. However, obtaining a history may be difficult as the constituents of many traditional medicines are often tightly guarded secrets not shared with patients or third parties. Laboratory analyses for cardiac glycosides are available, and an immunoassay developed for the detection of digoxin also potentially cross-reacts with other cardiac glycosides, such as oleandrin. However, more specific tissue and biological fluid assays for oleandrin have been developed.^8^

Of further interest has been the roles of other African plant materials which have been claimed to produce antihypertensive as well as negative inotropic and chronotropic effects.^9^-^11^ While some of the claims made for these plant materials can be supported by animal studies, more rigorous preclinical, clinical and toxicity (including cardiotoxicity) studies will have to be undertaken.

Management of plant-intoxicated patients includes immediate discontinuation of further exposure to the toxic plant materials, administration of activated charcoal and gastric lavage (caveat: within one hour of ingestion) and monitoring for dysrhythmias. Digoxin-specific antibody fragments appear to cross-react with at least some other cardiac glycosides, and therefore have a potential application in the treatment of poisoning in humans with the latter phytochemicals.^8^ If dysrhythmias are present, appropriate intervention and general supportive measures should be instituted. However, the mainstays of protection against the potentially toxic effects of plant materials remain in the educational realm and the prevention of ingestion.
